# Comparison between microcatheter and nebulizer for generating Pressurized IntraPeritoneal Aerosol Chemotherapy (PIPAC)

**DOI:** 10.1007/s00464-020-07546-z

**Published:** 2020-04-20

**Authors:** Laura Toussaint, Yaroslav Sautkin, Barbara Illing, Frank-Jürgen Weinreich, Giorgi Nadiradze, Alfred Königsrainer, Dörte Wichmann

**Affiliations:** 1 National Center for Pleura and Peritoneum, University Hospital Tübingen, Tübingen, Germany; 2 Department of Gastrointestinal Surgery, University Hospital Lausanne, Lausanne, Switzerland; 3 Institute of Pathology and Neuropathology, University Hospital Tübingen, Tübingen, Germany; 4 Department of General and Transplant Surgery, University Hospital Tübingen, Tübingen, Germany; 5 Interdisciplinary Endoscopy Unit, University Hospital Tübingen, Hoppe-Seyler Str. 3, 72076 Tübingen, Germany

**Keywords:** Intraperitoneal chemotherapy, Aerosol, Doxorubicin, Cisplatin, Peritoneal metastasis, Medical devices, PIPAC

## Abstract

**Background:**

This study compares an endoscopic microcatheter and a nebulizer for delivering Pressurized IntraPeritoneal Aerosol Chemotherapy (PIPAC).

**Methods:**

This is an in vitro and ex vivo study in an established model (inverted bovine urinary bladder). Four parameters were compared to determine the performance of a micro-perforated endoscopic spray catheter vs. state-of-the art, nozzle technology: (1) surface coverage and pattern with methylene blue on blotting paper at three different distances; (2) median aerodynamic diameter (MAD) of aerosol droplets with three different solutions (H_2_O, Glc 5% and silicon oil); (3) depth of tissue penetration of doxorubicin (DOX) and (4) tissue concentration of cisplatin (CIS) and DOX using standard clinical solutions.

**Results:**

The spray area covered by the microcatheter was larger (*p* < 0.001) but its pattern was inhomogenous than with the nozzle technology. We found that aerosol droplets were larger in the test group than in the control group for all three solutions tested. Median tissue penetration of DOX was lower (980 µm) with the microcatheter than with the nebulizer (1235 µm) and distribution was more heterogeneous ( = 0.003) with the microcatheter. The median tissue concentration of DOX and CIS was lower and concentration of DOX was more heterogeneous with the microcatheter (*p* = 0.002).

**Conclusions:**

This investigation has revealed that microcatheter technology generates larger aerosol droplet size, less drug tissue penetration and lower drug tissue concentration than the current nozzle technology. In the absence of clinical studies, use of microcatheters for delivering PIPAC can not be recommended at this stage.

Peritoneal metastasis (PM) has a poor prognosis and remains one of the great remaining challenges in modern oncology. Pressurized intra-peritoneal aerosol chemotherapy (PIPAC) has recently been introduced as a palliative option for treating PM and has shown encouraging clinical results [1]. Superior pharmacological properties of PIPAC have been demonstrated in preclinical models [2, 3] and clinical settings [4–6]. The current technology for generating PIPAC aerosols is a, conical single-fluid nozzle driven by an industry-standard high-pressure angioinjector [7]. This CE-certified technology is able to aerosolize a large range of substances, including chemotherapy solutions [8–10], nanomolecules [11, 12], and genes (DNA [4], RNA [13]). Although is recognized that current PIPAC technology achieves high drug concentrations in target tissues, limitations in the homogeneity of spatial distributions of the aerosol have been documented [14]. For physical reasons, the current droplet size of PIPAC aerosols (approximately 25 µm) might prevent significant improvement of this spatial distribution [15].

Khosrawipour et al. [16] suggested in a current publication, that PIPAC can be delivered via a spray catheter used for endochromography. Depth of doxorubicin (DOX) penetration into normal rodent peritoneal tissue was measured between 84 ± 45 and 348 ± 47 μm. The authors concluded that microcatheter spray technology was equivalent to current PIPAC nozzle technology [16], however, in that study, there was no control group with conventional nozzle technology and no granulometric data on the aerosol. Only a single-aqueous solution with low viscosity was tested, and sticky not a viscous or oily solution. Finally, no information on tissue drug concentration was provided. In the present study, we repeated and expanded the experiments reported above. For this purpose, we performed a comprehensive technical and pharmaceutical comparison between the endoscopic microcatheter and a catheter with the current nozzle technology, hereinafter called an advanced nebulizer, and used an in vitro and an ex vivo model to MAD sizes, surface coverage, drug penetration, and concentration in the tissue. Aim of this study was to clarify the effective principles for the treated tissue of both catheter devices.

## Materials and methods

### Design

This study used in-vitro and ex-vivo experiments to compare the performance of two devices for aerosolizing chemotherapy: (a) a test group with a spray microcatheter designed for endoscopic applications (PW-205V, Olympus, Hamburg, Germany); and (b) a control group with an advanced nebulizer (CapnoPen^®^, Capnomed, Zimmern, Germany) certified for aerosolization of solutions into body cavities. We analyzed four parameters: homogeneity of aerosol spatial distribution, granulometric results of the aerosol, depth of tissue drug penetration and tissue concentration of two chemotherapeutic drugs (DOX and cisplatin (CIS)).

### Sample size

Sample size was calculated on the basis of the data of Khosrawipour et al. [16] assuming a depth of tissue penetration for DOX of 348 ± 47 μm and considering that a difference of < 20% between devices would not be meaningful clinically. Further assumptions were an alpha-error of 0.05 and a power of 0.8. Using an online sample size calculator (stats/SampleSize.aspx, visited on Oct 17, 2019) a sample size of seven biopsies per group was determined. To account for possible sample drop-outs, nine biopsies in three bladders were performed.

### Ethical and regulatory background

No live animals were used or sacrificed for these experiments. The bovine bladders used in this study were obtained from the slaughterhouse from animals sacrificed for the alimentary chain. No human-derived specimens were used. Thus, according to German law, no authorization of the Institutional Review Board or of the Animal Protection Committee was required.

### Health and safety

The *ex vivo* experiments performed in this study involved the cytotoxic drugs CIS (Cisplatin Teva^®^, Teva, Ulm, Germany) and DOX (Doxorubicin HCl Teva^®^, Teva, Ulm, Germany), which are very toxic and present a potential hazard for the personnel involved. A risk analysis was performed and standard operating procedures were developed. All personnel involved were trained in these procedures. All experiments were performed in a class-3 safety hood certified for manipulating cytostatic drugs. Air measurements were performed under real conditions in the lab by an external, independent institution (DEKRA, Stuttgart, Germany), and platin contamination was prevented. The safety of the NCPP laboratory was audited successfully by Unfallkasse Baden-Württemberg, Stuttgart, Germany in Fall 2016.

### Spray coverage

For evaluating the area covered by the device, 25 ml of methylene blue (Methylene blue hydrate, Sigma-Aldrich Chemie GmbH; Steinheim; Germany) was sprayed vertically (downwards) onto a 60 × 60 cm blotting paper, at three different distances (5 cm, 10 cm and 15 cm). Paper was dried at room temperature (20 °C–24 °C). Then, high-definition pictures of the blots were taken at a distance of 30 cm under standard light and exposure conditions. Images were mounted using Photoshop software (Adobe Inc., San Jose, CA, USA) without any image processing. The contrast of the mounted image was optimized once with the same parameters for all panels.

### Granulometric measurements of aerosol particles

Granulometric measurements of aerosol particles were performed by laser diffraction spectrometry (Spraytec^®^; Malvern, Herrenberg, Germany). Three solutions with different viscosities were tested: distilled water (H_2_O), glucose 5% (Glc 5%), and silicon oil. All devices were driven by a high-pressure angioinjector (Accutron HP-D; Medtron AG, Saarbrücken, Germany) with the following settings: upstream pressure 11–19 bar, injection flow 0.5 ml/sec, volume 80 ml.

### Ex vivo model

The ex-vivo model used has been described previously [17]. Fresh bovine urinary bladders were obtained from the slaughterhouse. Since the bovine bladder is intraperitoneal and completely covered by peritoneum, inverting the organ provides a closed cavity covered with homogeneous serosa and a volume similar to that of the expanded human abdominal cavity (2–5 L). Fresh bladders were provided in the early morning and kept at a temperature of 4–8 °C. The bladders were prepared, cleaned, and inverted. A trocar (Kii^®^; Applied Medial, Düsseldorf, Germany) was inserted tightly into the bladder neck and a capno-peritoneum of 12–15 mmHg at room temperature (20 °C–24 °C) was established. Then, 2.7 mg DOX in 50 ml NaCl 0.9% and 13.5 mg CIS in 150 ml NaCl 0.9% were aerosolized. The system was kept in a steady-state for 30 min. After safe desufflation of the toxic aerosol though HEPA filters, the procedure was terminated and the bladder opened.

### Biopsies

All experiments were performed in triplicate, and involved 486 single measurements for depth of tissue penetration and 108 single measurements of drug tissue concentration. Details are provided in Table 1.

**Table 1:** Suppl. material

Device	*N* bladders	*N* biopsies	Depth of tissue penetration			Tissue concentration		
			*N* sections	*N* slides	*N* measurements	*N* probes (CIS)	*N* probes (DOX)	Total
Microcatheter	3	2 × 9	3/biopsy	3/section	3/slide	27	27	54
Nebulizer	3	2 × 9	3/biopsy	3/section	3/slide	27	27	54
Total	6	2 × 18	54	162	486	54	54	108

Bunch biopsies (8 mm) were performed in triplicate at three levels (top, middle, bottom) of the enhanced inverted bovine urinary bladder for measurements of tissue penetration (n = 9) and tissue concentration (n = 9). Biopsies were directional and placed onto a colored paper to exclude mistakes in orientation. All samples were immediately frozen and stored at − 80 °C.

### Drug concentration measurement

Biopsies were allowed to thaw at room temperature (RT) under a cytostatic hood. The biopsies were transferred into labeled 2-ml vials and kept at + 4° C in a fridge before lyophilization. Then, vials were placed into in a Speedvac device (S-Concentrator, BA-VC-300H; H. Saur, Laborbedarf, Reutlingen, Germany) and centrifuged under vacuum overnight (1000 rpm; 100 mbar) at RT. The dry pellets were weighed on a high accuracy scale (R180D; Sartorius, Germany) for later normalization. Then, the dry pellets were rehydrated with 1.5 ml of sterile distilled water (Ampuwa, Fresenius KABI, Homburg, Germany) and homogenized using a homogenizer (TissueLyser LT; QIAGEN GmbH, Hilden, Germany). Shortly after, the sample material and ceramic beads were placed together into 2 ml ceramic tubes (Ceramic Bead Tubes Kit; QIAGEN GmbH, Hilden, Germany) and shaken in a vertical position (50 Hz, 3000 oscillations/min) for 1 h at RT. Then, the tubes were placed into an ultrasounication device (Elmasonic S30H; Singen, Germany) for 10 min at RT. Finally, the tubes were mixed on a vortex mixer for 30 s, centrifuged for 10 min (5417R, 9000 rpm; Eppendorf, Hamburg, Germany) at RT and stored at − 80 °C.

### Drug concentration measurements

The tubes were shipped on dry ice to an external, GLP-certified laboratory (MVZ Dr. Eberhard & Partner Dortmund (ÜBAG), Dortmund, Germany). Biopsies were sent to an independent, GLP-certified laboratory (Labor Eberhard, Dortmund, Germany). The laboratory was blinded to the sample identities. The DOX concentration was measured by high-performance liquid chromatography (Waters Fluorescence Detector 2475; Waters Inc., Milford, MA), with a serum lower limit of quantification (LLoQ) of 5 ng/ml. Pre-analytical validation proved a linear range of measurements in Glc 5% matrix from 0.1 to 10,000 µg/ml DOX and established no interference by the organic matrices. The CIS was quantified by atomic absorption spectroscopy (ZEEnit P 650; Analytic Jena AG, Jena, Germany). The LLoQ for platinum was 50 ng/ml (CIS 80 ng/ml; calculation factor 1.54). Pre-analytical validation proved a linear range of measurements in Glc 5% matrix from 0.1 to 100 µg/ml platinum and established no interferences by the of organic matrices.

### Drug depth penetration

Samples from 3 × 3 biopsies (top, middle, bottom) were prepared for cryosection (Tissue-Tek, Sacura). Three 10 µm thick sections of each biopsy were cut at right angles to the surface of the punch biopsy (KT − 20 °C, OT − 21 °C). The sections were fixed with Cytoseal-xyl^®^ on a glass slide and covered. Then, the sections were air-dried at RT and analyzed. Measurements were performed using a fluoresecence microscope (DMRBE; Leica, Wetzlar, Germany) with Leica Qwin 2002 software after initialization and standardization. A picture at a magnification of 2.5× was taken to first provide an overview of the sample (size, morphology, completeness of anatomical layers, orientation). Nuclear fluorescence at an emission wavelength of 490 nm and absorption wavelength from 560 to 590 nm were used to determined depth of tissue penetration of DOX. Measurements were performed in triplicate by a trained biologist who was previously trained by a pathologist and blinded to the identity of the samples.

### Statistical analyses

**Fig. 1:**
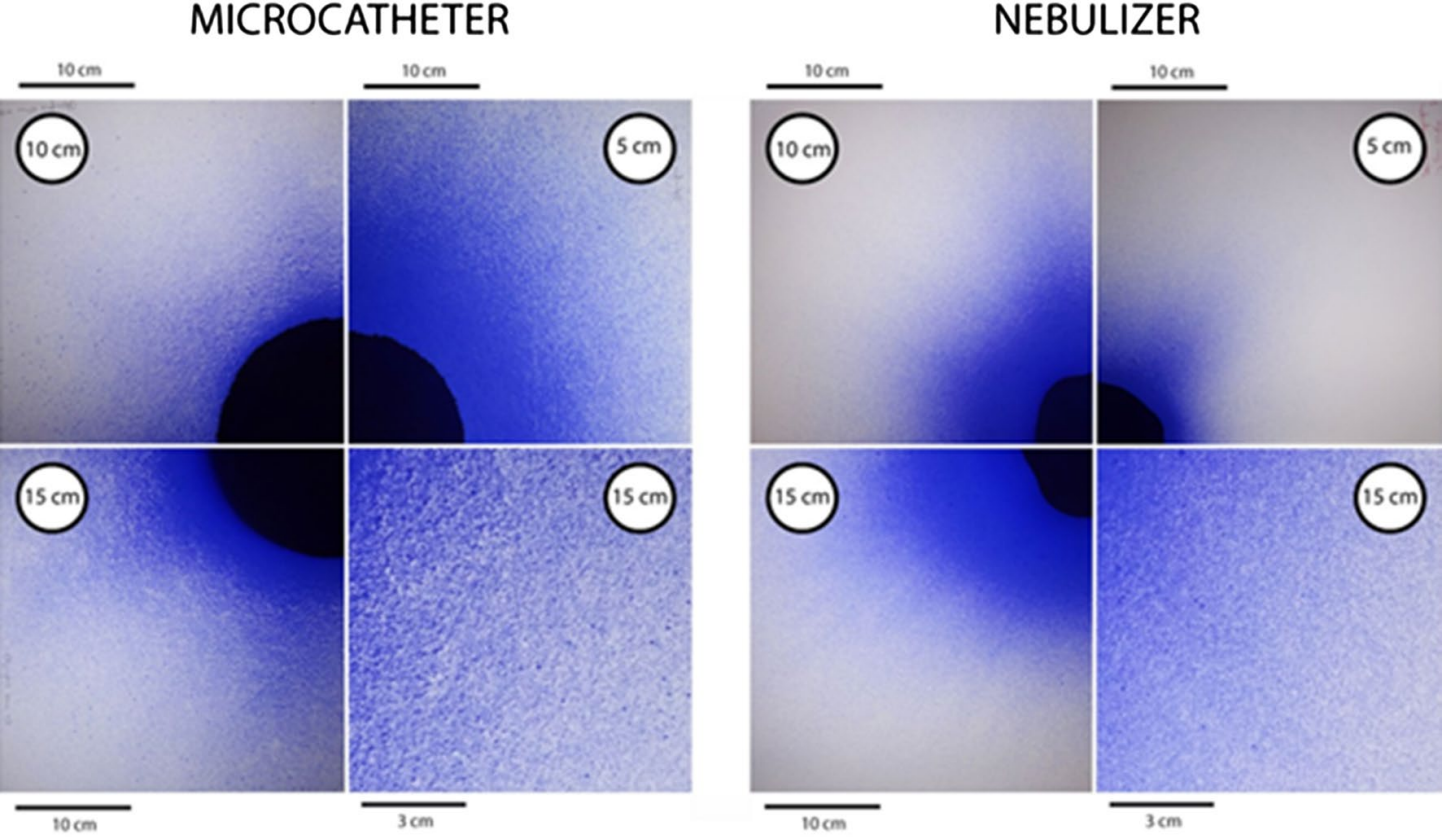
Comparison of the spraying patterns of the microcatheter (left panel) vs. nebulizer (right panel). Ink coverage (methylene blue) of a blotting paper placed at 5 cm (right upper panels), 10 cm (left upper panels) and 15 cm (left lower panels. Right lower panels: macroscopic view of a 100 cm^2^ central surface of the blot, showing a coarse, irregular pattern after spraying with a microcatheter

Descriptive statistics: Continuous data are expressed as the mean and confidence intervals 5%–95% or, when meaningful, as median values. Means between groups were compared by the Mann–Whitney *U* test or repeated variance analysis (ANOVA) performed in SPSS software v. 25 (IBM Corp., Armonk, NY, USA) (Fig. 1).

## Results

Results for the homogeneity of aerosol spatial distribution, granulometry of the aerosol, depth of tissue drug penetration, and tissue concentration of the two chemotherapeutic drugs (DOX and CIS), are summarized in Table 2.

**Table 2:** Comparison of spray coverage pattern, aerosol droplet size, depth of tissue penetration and tissue concentration between two devices: (a) a microperforated spray catheter certified for endoscopic applications (PW-205V, Olympus, Hamburg, Germany) and (b) an advanced nebulizer certified for intraperitoneal delivery of solutions (Capnopen^®^, Capnomed, Zimmern, Germany)

	Microcatheter (test)	Significance within group	Nebulizer (control)	Significance within group	Significance between groups
Median aerodynamic diameter (MAD) (µm; CI 10–90%)					
Water	56.2 (27.1–118.7)		34.8 (22.8–52.7)		N/A
Glc 5%	57.8 (23.4–120.3)		39.0 (23.7–65.2)		
Oil	48.2 (26.3–90.9)		43.0 (20.2–78.5)		
Depth of tissue penetration (DOX) (µm, mean ± STDV)					
Top	314.1 (44.9–583.2)	*p* = 0.03	1902.5 (833.7–2971.3)	*p* = 0.22	*p* = 0.29
Middle	655.3 (− 162.7–1473.6)		839.2 (91.6–1586.9)		
Bottom	1849.5 (390.3–3308.7)		1222.5 (273.1–2171.8)		
Blotting paper (mean, CI 5–95%)					
Distance from the paper (cm)	Inner diameter	*p* = 0.24	Inner diameter	*p* = 0.68	*p* ≤ 0.001
5	18.5 (17.2–19.7)		12.2 (11.3–13.1)		
10	20.1 (15.6–24.6)		12.5 (11.5–13.6)		
15	23.1 (10.7–35.7)		12.3 (10.9–13.8)		
Distance from the paper (cm)	Outer diameter	*p* ≤ 0.001	Outer diameter	*p* = 0.003	*p* ≤ 0.001
5	37.0 (34.5–39.5)		15.7 (14.2–17.2)		
10	28.7 (23.5–33.8)		19.0 (16.5–21.5)		
15	25.7 (24.2–27.1)		22.0 (17.0–27.0)		
Quality	Coarse, inhomogeneous		Fine, homogeneous		
Tissue concentration (DOX) (ng/mg, mean ± STDV)					
Top	0.8 (− 1.0–2.5)	*p* = 0.002	4.6 (1.6–7.5)	*p* = 0.30	*p* = 0.07
Middle	3.1 (− 2.7–8.9)		3.4 (0.02–6.8)		
Bottom	18.5 (7.3–29.7)		6.5 (3.2–9.8)		
Tissue concentration (CIS) (ng/mg, mean ± STDV)					
Top	63.3 (3.2–123.5)	*p* = 0.33	162.2 (102.8–221.6)	*p* = 0.03	*p* = 0.01
Middle	71.1 (18.7–123.5)		157.8 (97.7–217.9)		
Bottom	149.4 (74.3–229.0)		251.1 (186.2–316.0)		

Two drugs were tested: cisplatin (CIS) and doxorubicin (DOX)

### Coverage area and pattern

The surface stained with methylene blue was larger in the test group (microcatheter) than in the control (nebulizer) group, with a diameter ratio between 152 to 187%, depending on spray distance (p < 0.001)), whereas in the test group, this diameter decreased by 44% (p < 0.001), which suggested a short flying distance of the aerosol. The droplets are larger with an inhomogeneous shape in the test group.

### Granulometry

**Fig. 2:**
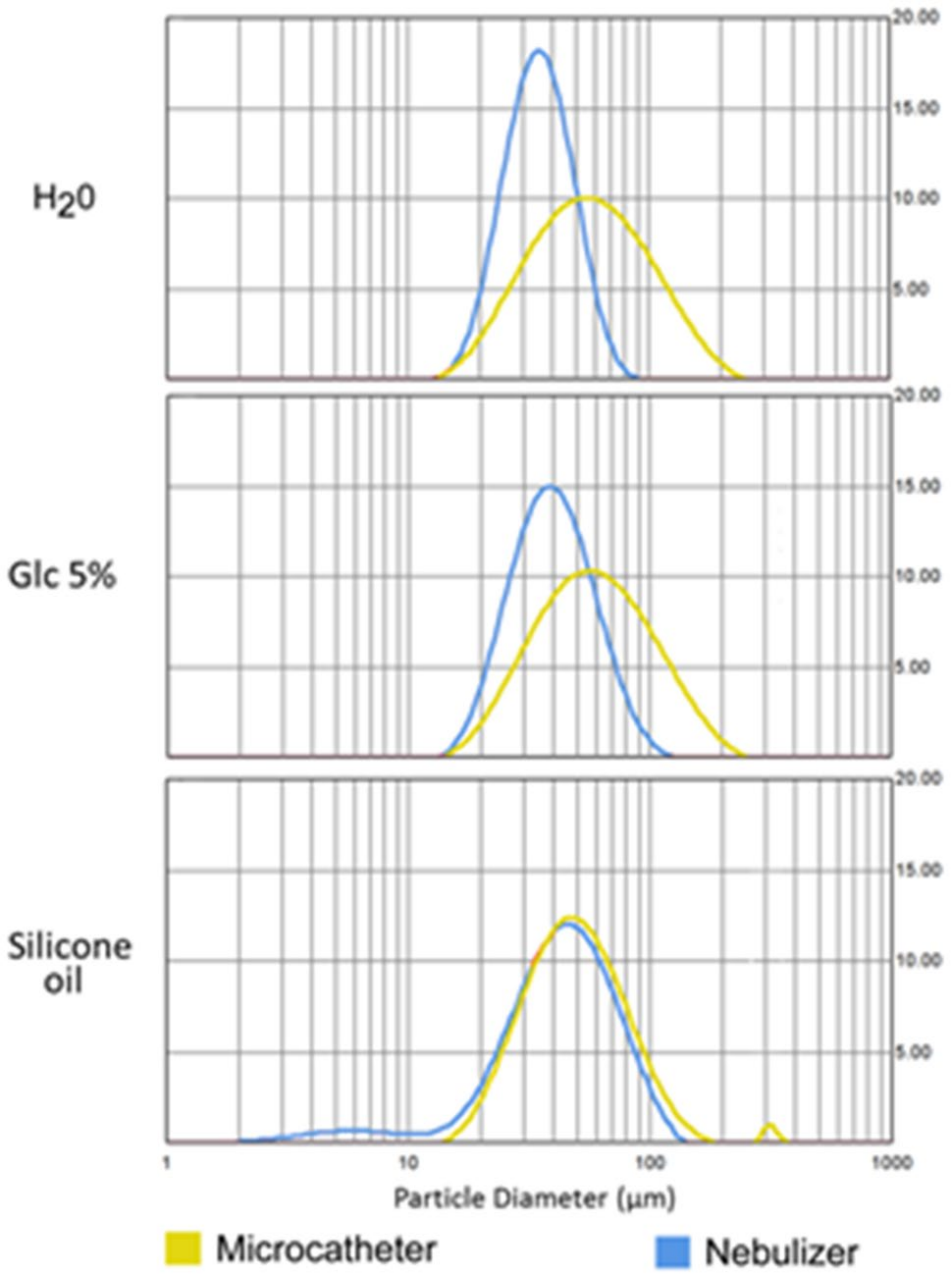
Comparison of the Median Aerodynamic Diameter (MAD) of the aerosol droplets after application with a spray microcatheter (yellow curves) or a nebulizer (blue curves). Three substances were tested: distilled water (H_2_O), Glc 5% and silicon oil. For aqueous or highly diluted solutions, MAD was larger with the spray catheter, and the size distribution was more heterogeneous (Color figure online)

The median aerosol diameter (MAD) of the aerosol droplets was measured for three typical substances: distilled water (H_2_O), Glc 5%, and silicon oil. The MAD was 61% larger after aerosolization of distilled water with the spray catheter (test) than with the nebulizer (control). To a lesser degree, this remained true for Glc 5% and silicon oil. For example, when Glc 5%, a solution used in clinical practice, the MAD was 58 µm [largest droplets (95% CI): 120 µm] in the test group vs. 39 µm [largest droplets (95% CI): 65.2 µm] in the control group. As shown in Fig. 2, the size distribution was more homogeneous in the control group (nebulizer) then in the test group (spray catheter). After aerosolization of silicon oil, a bimodal distribution was observed in both groups, with the appearance of droplets < 10 µm in the control group (nebulizer) and of droplets > 300 µm in the test group (spray catheter).

### Depth of tissue drug penetration

Among all measurements, the depth of tissue penetration of DOX was higher in the control group (median: 1235 µm) than in the test group (median: 980 µm). Spatial distribution was more homogeneous in the control group: in the test group, a significant gradient (0 = 0.003) was observed with a depth of tissue penetration that was six times shorter at the top than at the bottom in the ex-vivo model, representing a vertical distance of 25 cm. In contrast, there was no statistically significant gradient in the control group (*p* = 0.22).

### Drug concentration in tissue

**Fig. 3:**
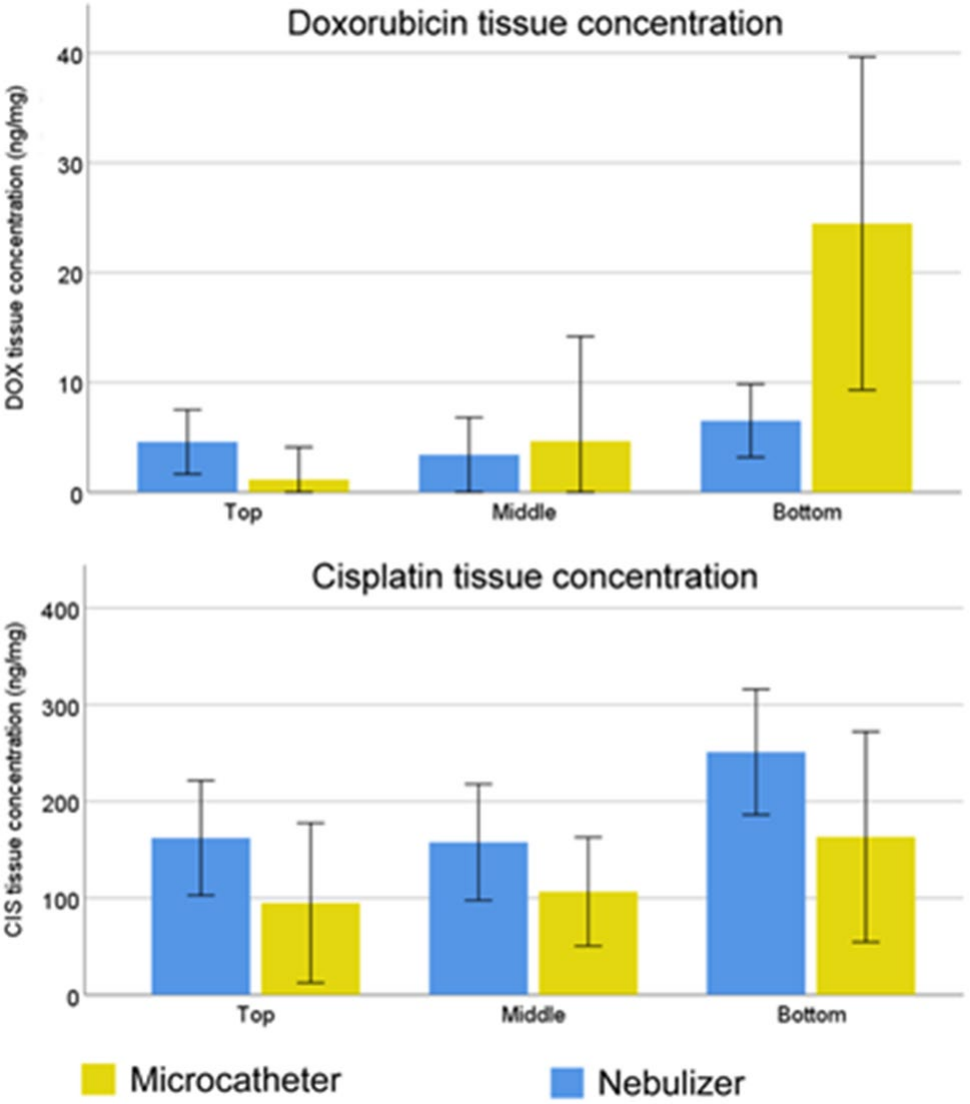
Comparison of tissue concentration of DOX and CIS between the test group (microcatheter) and the control group (nebulizer). For all measurements together, median tissue concentration is higher with the nebulizer both for DOX (*p* = 0.07) and CIS (*p* = 0.01)

**Fig. 4:**
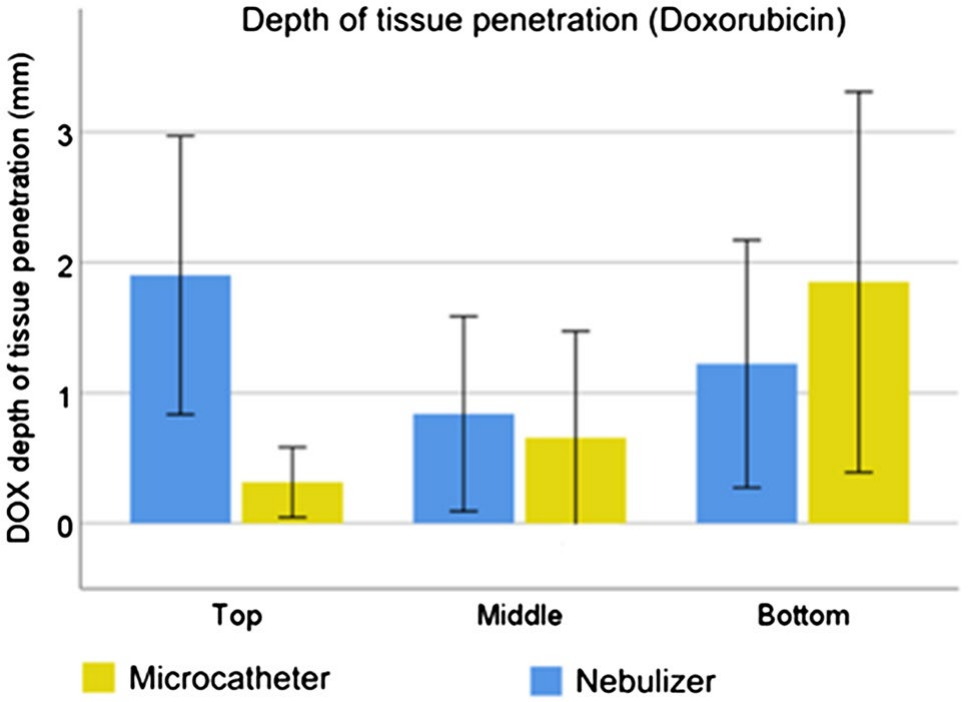
Depth of tissue of doxorubicin (DOX) as measured by fluorescence microscopy (nuclear staining) by an independent biologist blinded to the origin of samples. Homogeneity of spatial distribution after aerosolization with a nebulizer is superior to the performance of a spray microcatheter

Two drugs were tested: CIS and DOX. Among all experiments, the tissue concentration of DOX was higher in the control group (median: 5.6 ng/mg tissue) than in the test group (median: 2.6 ng/mg tissue) but the difference was not significant (*p* = 0.07). The spatial distribution was more heterogenous in the test group (microcatheter, *p* = 0.002) than in the control group (nebulizer, *p* = NS). Taken together, the tissue concentration measurements of DOX (Fig. 3, upper panel) largely confirmed the depth of penetration data (Fig. 4) for both tested devices. Similar results were obtained with CIS, for which the tissue concentration was higher in the control group (185 ng/mg) than in the test group (125 ng/mg) (*p* = 0.01) among all experiments.

## Discussion

PIPAC is an innovative promising delivery system for intraperitoneal drug delivery that has been shown to be feasible and safe. Data on objective responses and quality of life of patients have been encouraging. Therefore, PIPAC can be considered as a treatment option for refractory isolated PM of various origins [1]. However, this technology is still in its infancy, so further optimization is needed to exploit its full potential [15].

Khosrawipour et al. [16] proposed the use of a microcatheter instead of the nebulizer for all published PIPAC clinical studies so far. This microcatheter was designed for endoscopic spraying of staining solutions onto intraluminal lesions [18]. The authors found that the mean depth of DOX penetration was between 84 and 348 μm and significantly higher in tissue directly exposed to the aerosol jet. All samples had contact with DOX. Those authors concluded that local drug penetration using a microcatheter was practically congruent with known PIPAC performance of nebulizer and claimed that compared with conventional PIPAC, the microcatheter offered better feasibility and flexibility, easier handling, and lower cost.

However, and somewhat surprisingly, that study had no control group and no quantitative comparative data were provided. Only a single aqueous solution and a single drug (DOX) were tested. No granulometric measurements, no spray coverage data and no tissue concentration measurements were provided.

For our comparative study, microcatheters with and without nebulizer technology were tested by analyzing four parameters: granulometry of the aerosol, depth of tissue drug penetration of doxorubicin, tissue concentration of two chemotherapeutic drugs (DOX and CIS), and homogeneity of the aerosol spatial distribution. We measured a larger aerosol droplet size in the test group than in the control group for all three tested solutions. The area covered by the spray was larger but heterogeneous in the test group. Median tissue penetration of DOX was lower and the distribution was more heterogeneous in the test group. The median tissue concentration of DOX and CIS was lower and concentration of DOX was more heterogeneous in the test group. In the present study, data for tissue concentration and depth of tissue penetration of doxorubicin are congruent. Thus, we can not confirm, that local penetration of DOX by using an endoscopic microcatheter without nebulizer technology was practically congruent with known PIPAC performance with the nebulizer. We found that the in-vitro performance and target effect on the tissue of an advanced nebulizer were clearly superior to those of the microcatheter technology for three of four of the tested parameters. The meaning of the larger impaction zone of the aerosol and the inhomogeneous spray pattern is not clear at present.

We allow that further developments of PIPAC technology might include micro-perforated catheters for selected indications and solutions. However, on the basis of our experimental findings and in the absence of clinical validations of the claims of Khosrawipour et al. [16], at the present stage we cannot recommend the use of endoscopic microcatheters for delivering chemotherapeutic drugs as aerosols under pressure into the abdominal cavity of human patients.

Conclusively: Our results show that micro-perforated endoscopic spray catheters generate larger aerosol droplet size, broader coverage surface, less drug tissue penetration, lower drug tissue concentration and more inhomogeneous spatial distribution than the advanced nebulizer. On the basis of our experimental findings and in the absence of clinical validations of the claims of Khosrawipour et al. [16], we cannot recommend the use of endoscopic microcatheters for delivering chemotherapeutic drugs as aerosols under pressure into the abdominal cavity of human patients at the present developmental stage.

## Abbreviations

^®^: Registered trademark
CIS: Cisplatine
Control group: CapnoPen^®^ nebulizer
DOX: Doxorubicine
IBUB: Inverted bovine urinary bladder
IPC: Intra peritoneal chemotherapy
MAD: Median absolute deviation
Ns: Non-significant
PIPAC: Pressurized Intra-Peritoneal Chemotherapy
PM: Peritoneal metastases
RT: Room temperature
SPSS: Statistical Package for the Social Sciences
Test group: Olympus^®^ microcatheter
TL: Tissue Lyser
