# Correction to: Comparison between microcatheter and nebulizer for generating Pressurized IntraPeritoneal Aerosol Chemotherapy (PIPAC)

**DOI:** 10.1007/s00464-021-08577-w

**Published:** 2021-06-09

**Authors:** Laura Toussaint, Yaroslav Sautkin, Barbara Illing, Frank‑Jürgen Weinreich, Giorgi Nadiradze, Alfred Königsrainer, Dörte Wichmann

**Affiliations:** 1 National Center for Pleura and Peritoneum, University Hospital Tübingen, Tübingen, Germany; 2 Department of Gastrointestinal Surgery, University Hospital Lausanne, Lausanne, Switzerland; 3 Institute of Pathology and Neuropathology, University Hospital Tübingen, Tübingen, Germany; 4 Department of General and Transplant Surgery, University Hospital Tübingen, Tübingen, Germany; 5 Interdisciplinary Endoscopy Unit, University Hospital Tübingen, Hoppe-Seyler Str. 3, 72076 Tübingen, Germany

## Correction to: Surgical Endoscopy (2021) 35:1636–1643 https://doi.org/10.1007/s00464-020-07546-z

The article “Comparison between microcatheter and nebulizer for generating Pressurized IntraPeritoneal Aerosol Chemotherapy (PIPAC),” written by Laura Toussaint, Yaroslav Sautkin, Barbara Illing, Frank-Jürgen Weinreich, Giorgi Nadiradze, Alfred Königsrainer, and Dörte Wichmann, was originally published electronically on the publisher’s internet portal on 28 April 2020 without open access. With the author(s)’ decision to opt for Open Choice the copyright of the article changed on 20 May 2021 to © The Author(s) 2020 and the article is forthwith distributed under the terms of the Creative Commons Attribution 4.0 International License, which permits use, sharing, adaptation, distribution and reproduction in any medium or format, as long as you give appropriate credit to the original author(s) and the source, provide a link to the Creative Commons licence, and indicate if changes were made. The images or other third party material in this article are included in the article’s Creative Commons licence, unless indicated otherwise in a credit line to the material. If material is not included in the article’s Creative Commons licence and your intended use is not permitted by statutory regulation or exceeds the permitted use, you will need to obtain permission directly from the copyright holder. To view a copy of this licence, visit http://creativecommons.org/licenses/by/4.0

